# Fast frequency-sweep spectroscopic imaging with an ultra-low flip angle

**DOI:** 10.1038/srep30066

**Published:** 2016-07-21

**Authors:** Junyu Guo, Zoltan Patay, Wilburn E. Reddick

**Affiliations:** 1 Department of Diagnostic Imaging, St. Jude Children’s Research Hospital, Memphis, 38105 Tennessee, USA

## Abstract

Magnetic resonance (MR) spectroscopic imaging has become an important tool in clinical settings for noninvasively obtaining spatial and metabolic information on a molecular scale. Conventional spectroscopic imaging is acquired in the time domain, and its clinical application is limited by the long acquisition time, restricted spatial coverage, and complex suppression and reconstruction procedures. We introduce a fast MR spectroscopic imaging technique in the frequency domain, termed phase-cycled spectroscopic imaging (PCSI). PCSI uses a balanced steady-state free precession (bSSFP) sequence with an ultra-low flip angle to achieve very high acquisition efficiency with a short repetition time. This approach enables faster frequency sweeping by changing the cycled RF phase and using flexible non-uniform sampling, and it greatly reduces the RF energy deposition in tissue. With its intrinsic water and fat suppression, PCSI more closely resembles routine clinical scans because it eliminates the suppression steps. We demonstrate that it is feasible to acquire PCSI spectra in a phantom and in humans and that PCSI provides an efficient spectroscopic imaging method, even for J-coupled metabolites. PCSI may enable spectroscopic imaging to play a larger role in the clinical assessment of the spatial tissue distribution of metabolites.

Magnetic resonance spectroscopy (MRS) is widely used in medicine, biology, chemistry, and physics. Proton MRS has become an important tool in a broad range of clinical applications, enabling the noninvasive detection of subtle metabolic changes in tissues. As the spectrum is measured in either the frequency domain or the time domain, MRS techniques are categorized as either frequency-resolved or time-resolved techniques^1^,^2^,^3^. The early frequency-resolved techniques, which swept a range of frequencies by changing the magnetic field strength or radio frequency (RF), were very slow and could take several hours to acquire a single spectrum^1^,^2^. Therefore, these frequency-resolved techniques have now been supplanted by time-resolved techniques, which are faster and can offer much greater sensitivity^3^. For *in vivo* time-resolved spectroscopy, the signal has to be restricted to a selected volume in the subject. Surface coils were first used to receive the signal from a limited volume^4^. By using RF pulses and gradients, the targeted volume can be selected by different techniques^5^,^6^,^7^,^8^,^9^, such as PRESS (point-resolved spectroscopy)^9^, STEAM (stimulated echo acquisition mode)^8^, and ISIS (image-selected *in vivo* spectroscopy)^5^. These advances have made single-voxel spectroscopy (SVS) feasible in clinical settings.

Magnetic resonance spectroscopy imaging (MRSI) simultaneously acquires both metabolic and anatomic spatial information, thereby providing metabolic mapping that is of great interest to clinicians^10^,^11^. However, MRSI has not been widely accepted as a routine clinical tool, mainly because its data acquisition is very time consuming, and the post-processing procedures are generally performed offline, automatically or manually by an MR technician. For example, it may take more than 30 min to acquire an image with a 32 ×  32 matrix using the PRESS sequence. Multivoxel MRSI with an 8 × 8 or 16 × 16 matrix is used in routine clinical applications. In research studies, fast MRSI methods have been proposed for reducing the acquisition time^12^,^13^,^14^,^15^,^16^,^17^,^18^. In addition, the strong water signal overpowers the tiny metabolite signal (which is approximately 10,000 times weaker). Water suppression methods, such as chemical-shift selected pulses (CHESS)^19^ or MEGA^20^, are normally applied in MRSI. The lipid signal in the skull has to be suppressed to prevent it from contaminating the spectra obtained inside the brain. Lipid suppression can be achieved by several different strategies: outer volume suppression (OVS)^21^,^22^,^23^,^24^, the inversion recovery (IR) method^25^,^26^, or k-space data post-processing^27^. The most commonly used approach in clinical studies is OVS, which uses spatial presaturation bands to prevent spectral contamination by peripheral lipid and water signals^21^,^22^,^23^,^24^. The precise placement of the many OVS bands is challenging and time consuming, and it requires special technician training and skills.

We propose a novel frequency-resolved method called phase-cycled spectroscopic imaging (PCSI), which uses the bSSFP sequence with an ultra-low flip angle (less than 1°). This PCSI method may overcome the abovementioned problems to achieve very high acquisition efficiency with a short repetition time (TR). PCSI is the first pulsed frequency-resolved method; it uses RF pulses instead of continuous-wave RF as was used in the early frequency-resolved methods^1^,^2^. The bSSFP sequence with small flip angles (e.g., 4° or 5°) has been used for functional magnetic resonance imaging (fMRI)^28^,^29^, but the application of the bSSFP with ultra-low flip angles (<1°) has not been reported previously. The PCSI method uses ultra-small RF pulses and a phase-cycled frequency-sweep technique to make data acquisition faster, more efficient, and more flexible. Here, we demonstrate the feasibility of the PCSI method by conducting a conceptual validation with simulations and provide proof-of-principle testing in phantoms, volunteers, and a patient with a heterogeneous brain tumor. Our technique may provide a basis for developing new low-energy and high-sensitivity MRI techniques.

## Results

### Ultra-low flip angle steady state

**Figure 1:**
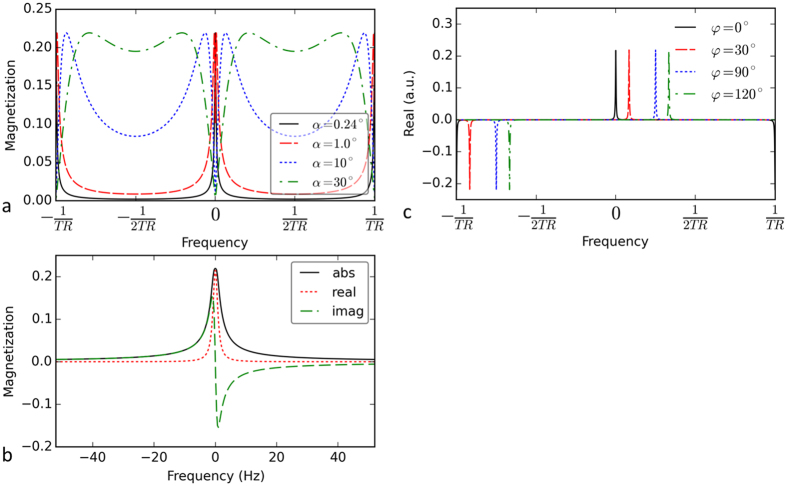
Simulated transverse magnetization profiles of a balanced SSFP sequence obtained using equation [S7]. (**a**) The magnitude profiles of four selected flip angles. (**b**) Enlarged magnetization profiles of the absolute, real, and imaginary components with *α* = 0.24°. (**c**) The real profiles shift along the frequency direction as the cycled RF phase *φ* is changed.

The magnetization profiles of the steady-state magnetization were generated for different flip angles, as shown in Fig. 1a. As the flip angle decreased to less than 1°, the profile became a single sharp peak. In Fig. 1b, the real part of the magnetization profile is a pure absorption shape and is much narrower than the magnitude profile. This real magnetization profile can serve as a perfect frequency response function for spectroscopic imaging. Using this response function, we can excite only a very narrow range of frequencies (approximately 1.9 Hz) and simultaneously suppress the signal at other frequencies. Therefore, we can acquire an MR image at a specific frequency point using this steady state with an ultra-low flip angle.

### Phase-cycled frequency sweeping

The peak locations of the response function are determined by the cycled RF phase *φ* in Fig. 1c. The cycled phase corresponds to a certain off-resonance frequency, Δ*f*, determined via the equation Δ*f* *=* *φ/*(2π · TR) derived from equation [S12] in the Supplemental Information. By changing the cycled RF phase, the peak location can be shifted to a target frequency within a frequency period, [−1/(2TR), 1/(2TR)], which corresponds to one RF phase cycle. Changing the cycled RF phase is much easier than changing the magnetic field, as was required in early frequency-resolved techniques. Taking advantage of the high acquisition efficiency of the bSSFP sequence, we obtained a PCSI sweep rate of approximately 13 images/s (or 76.8 ms per image), which is much more efficient than the early frequency-sweeping methods (which might have a sweep rate of approximately 1 Hz/s). This high sweep rate enables PCSI to acquire multiple averages in order to improve its signal-to-noise ratio (SNR). After obtaining a series of MR images at different frequency points, we can generate a spectrum in each voxel.

### Specific absorption rate

The ultra-small flip angle may greatly reduce the RF energy deposited in tissue, because the specific absorption rate (SAR) is proportional to the square of the flip angle, as follows:


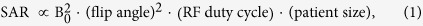


where the RF duty cycle is the ratio of the total RF duration time to the TR. There are three pulses (90°–180° –180°) in each TR (2000 ms) for the PRESS sequence, and one RF pulse (e.g., 0.3°) in each TR (2.4 ms) for the PCSI sequence. The SAR ratio of CSI to PCSI is computed to be 2916 by assuming the same duration for RF pulses. Additional RF pulses for the water- and lipid-suppression in CSI, if included, could further substantially increase this SAR ratio. The total RF energy of the PCSI sequence is 2.6 W·s, based on the reported protocol with a flip angle of 0.3°.

### Conceptual validation by simulation

**Figure 2:**
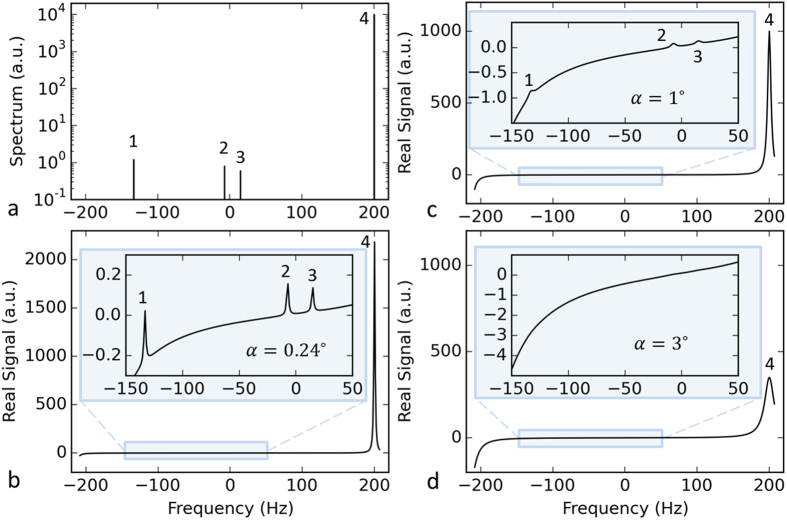
Simulation of a spectrum using the PCSI method. (**a**) The assumed metabolite signals of NAA (1), Cr (2), Cho (3), and water (4) on a log scale. (**b**) The simulated real signal obtained using equation [S7] with TR = 2.4 ms, *α* = 0.24°. The inset shows the enlarged plot with three metabolite peaks. (**c**) The simulated real signal with *α* = 1°. (**d**) The simulated real signal with *α* = 3°. In the simulations, the water peak was assumed to reside at 200 Hz.

We first assessed whether and under what conditions the extremely small metabolite signals can be obtained by using small RF pulses. We performed simulations with multiple small flip angles to validate the theoretical feasibility of PCSI and investigate the importance of the choice of RF flip angle. In the simulations, a spectrum was created with four peaks: water, choline (Cho), creatine (Cr), and N-acetyl aspartate (NAA), which were assumed to be delta functions with magnitudes of 10,000, 0.6, 0.8, and 1.2, respectively. Figure 2a shows the original spectrum with a water peak at 200 Hz. Figure 2b shows the simulated spectrum with an optimal flip angle, *α* = 0.24°, computed using equation [S11] with TR = 2.4 ms. This simulated spectrum was generated by convolution of the real response function in Fig. 1b and the spectrum in Fig. 2a. All three metabolic peaks could easily be observed (Fig. 2b), having heights proportional to the prescribed values. Figure 2c shows the simulated spectrum with *α* = 1°, in which the three small metabolic peaks are barely detectable. The three metabolic peaks are not appreciable in Fig. 2d with *α* = 3°. These simulations demonstrate that it is theoretically feasible to obtain a spectrum by using PCSI and that the metabolic peaks become smaller and eventually disappear as the flip angle increases. Therefore, an ultra-low flip angle is crucial if a spectrum is to be acquired by PCSI.

### Phantom study

**Figure 3:**
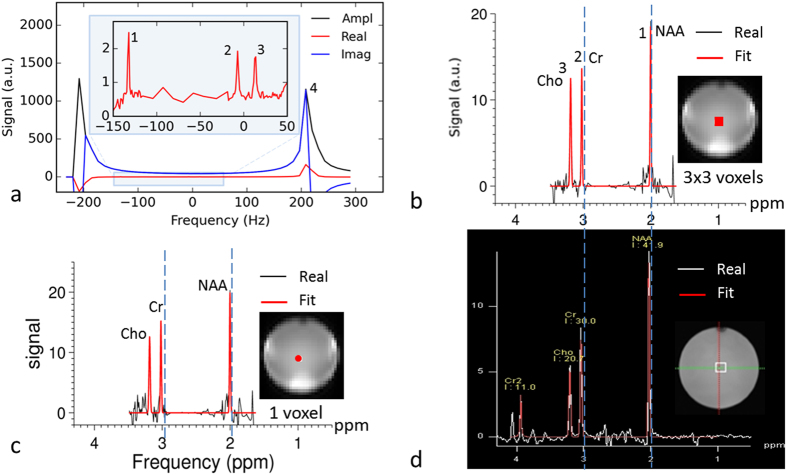
MR spectra from a phantom. (**a**) The PCSI spectral signal from nine voxels in the center (18.75 × 18.75 × 15 mm) with an inset showing three metabolite peaks. (**b**) The real and fitted PCSI spectra calculated from the spectral signal in (**a**), with the location shown in the inset. (**c**) PCSI spectra from a single voxel (6.25 × 6.25 × 15 mm) at the same location as (**b**) (shown in the inset). (**d**) Conventional spectra from a commercial single-voxel spectroscopy sequence; the voxel size is 20 × 20 × 20 mm (shown in the inset). The peaks labeled 1, 2, 3, and 4 are the NAA, Cr, Cho, and water peaks, respectively. Ampl, amplitude; Real, real part of the signal; Imag, imaginary part of the signal; Fit, the fitted spectrum. The inset images in (**b**) and (**c**) were generated from the PCSI data. The inset image in (**d**) was a high-resolution T_2_-weighted image at the same slice position shown in the insets in (**b**) and (**c**).

A proof-of-principle study was performed on a phantom filled with a brain-mimicking solution that contained the metabolites NAA, Cr, and Cho. PCSI data were acquired using a modified bSSFP sequence, in which the cycled RF phase was changed for each measurement by using a non-uniform frequency-sampling scheme. Figure 3a shows PCSI signals in a region of interest (ROI), including nine voxels (18.75 × 18.75 × 15 mm, or 5.3 cm^3^) at the center of the phantom. The peaks on either side are water peaks, which wrap around as a result of the periodic response function. Phase correction based on the water peaks was performed to convert the real component of each complex signal to a pure absorption shape. The inset enlargement in Fig. 3a clearly demonstrates the three metabolite peaks in the real signal. This signal can be converted to the spectrum, as shown in Fig. 3b after post-processing (see the Methods section for details). Figure 3c shows a spectrum from a single voxel (6.25 × 6.25 × 15 mm, or 0.59 cm^3^) at the same location as that in Fig. 3b. The single-voxel spectrum is similar to the spectrum in Fig. 3b from a larger ROI but has slightly more noise. For comparison, Fig. 3d shows a spectrum from a single voxel (20 × 20 × 20 mm, or 8 cm^3^) obtained by using the conventional time-resolved SVS sequence. The two spectra shown in Fig. 3b (PCSI) and 3d (SVS) were similar and had consistent peak positions. This phantom study demonstrates that PCSI can require much less time than the time-resolved SVS method to acquire comparable spectra.

**Figure 4:**
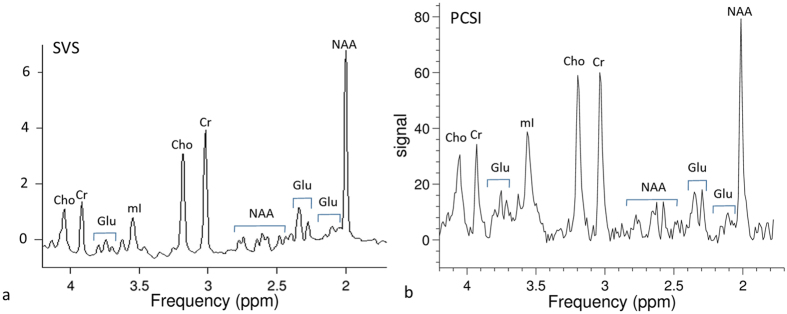
Spectra of a phantom obtained using single-voxel spectroscopy (SVS) and PCSI methods. (**a**) The SVS PRESS spectrum with a short TE of 30 ms; the voxel size was 20 × 20 × 20 mm. (**b**) The PCSI spectrum from an ROI with three voxels (each voxel was 6.25 × 6.25 × 15 mm). J-coupled metabolites, such as glutamate (Glu) and myo-inositol (mI), can be identified in both spectra.

**Figure 5:**
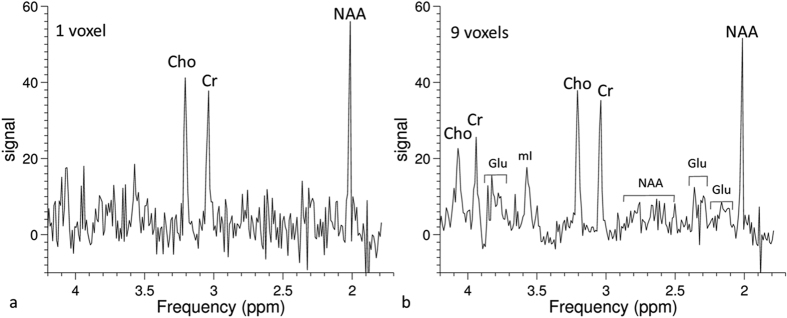
Phantom spectra from PCSI data with a 64 × 32 matrix. (**a**) The spectrum from one voxel (3.75 × 3.75 × 15 mm). (**b**) The spectrum from nine voxels (total dimensions 11.25 × 11.25 × 15 mm). The voxel in (**a**) is in the center of the ROI in (**b**).

Figure 4 shows a comparison between a short-TE SVS spectrum and a uniformly-sampled PCSI spectrum on the phantom. Small J-coupled metabolite signals, such as myo-inositol (mI) and glutamate (Glu), are clearly visible in both spectra. Figure 5 shows two spectra from a uniform PCSI with a 64 × 32 matrix and a higher in-plane resolution (3.75 × 3.75 mm). The metabolites with larger signals, such as NAA, Cr, and Cho, can be easily identified in the spectrum, even from a single voxel. In comparison, J-coupled metabolites, such as Glu and mI, can be identified in the averaged spectrum from nine voxels.

### Human studies

Spectroscopic imaging of the human brain is more challenging than phantom imaging. The field inhomogeneity of the human brain is greater than that of the phantom and can greatly diminish or even destroy metabolite signals. Also, the strong lipid signal in humans can degrade the quality of the spectrum. As a practical proof of principle of PCSI in human brain, volunteer and patient studies were performed using the same non-uniform–sampling protocol as was used in the phantom study.

**Figure 6:**
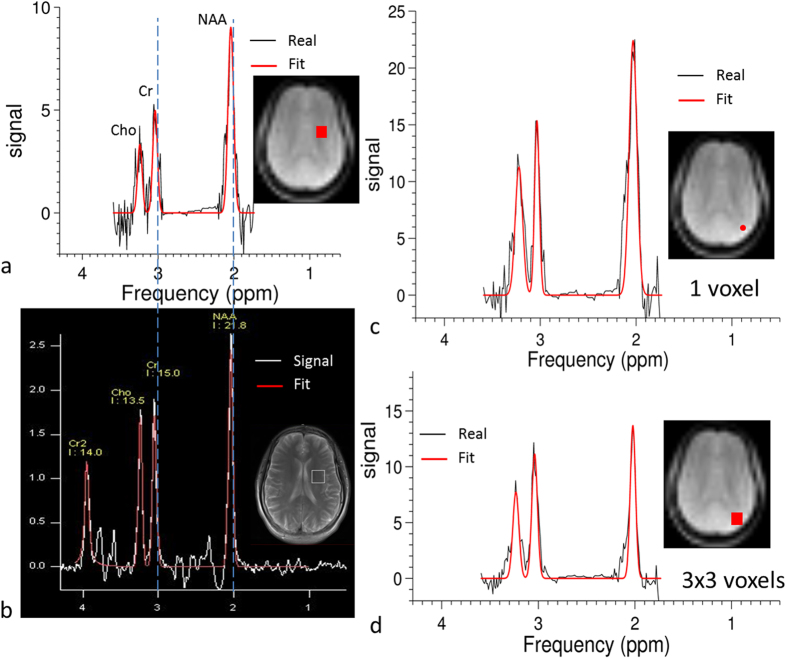
MR spectra from a healthy human volunteer. (**a**) PCSI spectra from the nine voxels (3 × 3 voxels with dimensions 18.75 × 18.75 × 15 mm) shown in red in the inset. (**b**) Conventional spectra from a commercial single-voxel spectroscopy sequence. The voxel size is 20 × 20 × 20 mm; the inset shows the ROI. (**c**) PCSI spectra from a single voxel (6.25 × 6.25 × 15 mm) at the periphery of the brain (shown in red in the inset). (**d**) PCSI spectra from nine voxels (3 × 3 voxels, with dimensions 18.75 × 18.75 × 15 mm) at the same location shown in the insert in (**c**). The inset images in (**a**,**c,d**) were generated from the PCSI data. The inset image in (**b**) was a high-resolution T_2_-weighted image acquired at the same slice position. ROI, region of interest; Real, real part of the signal; Fit, the fitted spectrum.

Figure 6a shows an *in vivo* PCSI spectrum from an ROI including 9 voxels (18.75  × 18.75 × 15 mm, or 5.3 cm^3^), in which three metabolite peaks can easily be identified and fitted. In comparison, a spectrum from a voxel (20 × 20 × 20 mm, or 8 cm^3^) obtained by using the SVS sequence is shown in Fig. 6b, in which the inset shows the location of the ROI to be similar to that in Fig. 6a. Both spectra show consistent peak positions and relative peak heights after alignment. Figure 6c shows a PCSI spectrum from a single voxel (6.25 × 6.25 ×15 mm, or 0.59 cm^3^) at the edge of the brain where field inhomogeneity becomes severe. Figure 6d shows a spectrum from an ROI at the same position that includes nine voxels (18.75 × 18.75 × 15 mm, or 5.3 cm^3^). Although the two spectra appear similar, the spectrum in the larger ROI has smaller peaks than does the single-voxel PCSI spectrum because of the high field inhomogeneity near the edge. Both spectra had less noise than did the spectrum in Fig. 6a, because the locations in Fig. 6c,d were closer to the head coil where the SNR is higher. The greater peak width in the PCSI spectra was mainly due to greater field inhomogeneity, because PCSI acquired data from a whole slice, whereas SVS acquired data from only a single voxel.

**Figure 7:**
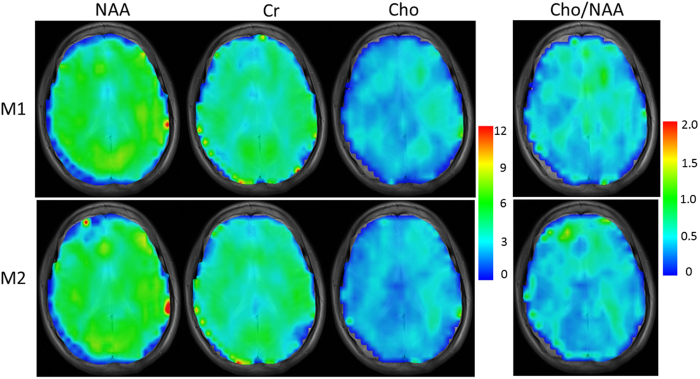
The normalized parametric maps of three metabolites (NAA, Cr, and Cho) and the parametric maps of the Cho/NAA ratio from two repeated measurements made using PCSI. M1 represents the first measurement; M2 represents the second measurement.

Parametric maps of the heights of the three-metabolite peaks and of the Cho/NAA ratio were generated for two repeated measurements, as shown in Fig. 7. The spectra were computed for each voxel (6.25 × 6.25 mm) by averaging the voxel and its four neighbors to improve its SNR. The two sets of maps were then interpolated and overlaid on a high-resolution T_2_-weighted image (voxel size: 0.52 × 0.52 mm) for visual comparison. The PCSI method demonstrates a good robustness for separate measurements.

**Figure 8:**
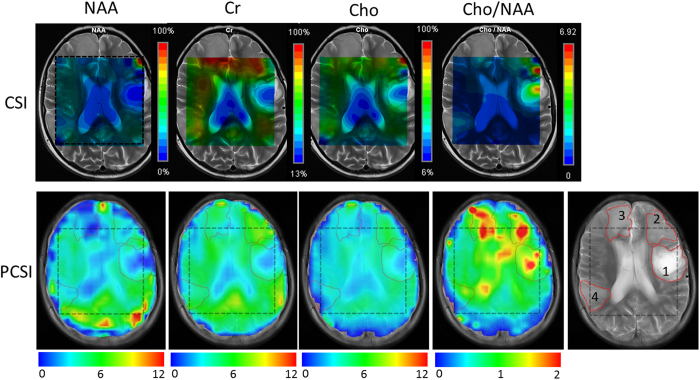
Parametric maps of three metabolites (NAA, Cr, and Cho) and of the Cho/NAA ratios from a patient with a multifocal anaplastic astrocytoma. Maps from the conventional CSI sequence with 10 × 10 voxels are in the upper row. The corresponding PCSI parametric maps are in the lower row. A T_2_-weighted image (far-right end of the lower row) shows the lesion foci (1–4); lesion 1 corresponds to a surgical cavity and lesions 2 through 4 represent tumor foci. The colored parametric maps were overlaid on a T_2_-weighted image. The dashed squares were manually drawn on PCSI maps and the T_2_-weighted image for comparison purposes.

As a final practical proof of principle, we compared the conventional chemical-shift imaging (CSI) parametric maps with PCSI maps for a patient with a multifocal anaplastic astrocytoma (a WHO grade 3 tumor) (Fig. 8). There were four lesion foci (1–4) visible in one T_2_-weighted image, in which lesion 1 corresponds to a surgical cavity and lesions 2 through 4 represent tumor foci. Figure 8 shows the importance of full coverage of the whole slice because of the heterogeneity of the tumors. It took approximately 4 min longer for CSI to acquire parametric maps with only 10 × 10 voxels than for PCSI to acquire maps with 32 × 32 voxels. The main tumor (lesion 1) can be clearly identified in the Cho/NAA ratio maps obtained with either method. However, the conventional CSI maps covered only small parts of lesions 2 and 3, which clearly demonstrate characteristic tumor profile features in the PCSI Cho/NAA map.

Because of the limited coverage of CSI maps, most lesion foci, including the left frontal surgical resection cavity (lesion 1) are inadequately evaluated. A slight increase in Cho is suggested in the portions of lesions 2 and 4 covered by the CSI maps. The corresponding PCSI parametric maps also show slightly increased Cho in lesions 2 and 3, decreased NAA concentrations in all lesion foci, and resultant robust increases of Cho/NAA in lesions 2 and 3 and a modest increase of Cho/NAA in lesion 4. The reason for the increased Cho/NAA ratio in lesion 1 is unclear; it may indicate residual or recurrent tumor, but this has not been confirmed.

## Discussion

We have demonstrated the PCSI method, a new, flexible, and efficient way to perform MR spectroscopic imaging in the frequency domain. PCSI uses a new frequency-sweep technique and a non-uniform sampling scheme that reduce the total acquisition time. PCSI also offers intrinsic water and fat suppression, thereby reducing operator dependence and greatly simplifying the scanning procedures. By using an ultra-low flip angle, PCSI greatly reduces the SAR in human subjects. In addition, PCSI may be suitable for measuring J-coupled metabolites because of its short effective TE and synchronized phases. PCSI has great potential to enable faster and simpler clinical applications of MRSI.

### Imaging speed

Conventional time-resolved MRSI is limited in its clinical applications because of the long acquisition times. Fast time-resolved MRSI methods have been proposed to accelerate the acquisition^12^,^13^,^14^,^15^,^30^,^31^,^32^,^33^,^34^. Of these methods, EPSI or PEPSI^12^,^14^, spiral MRSI^13^, and SI-CONCEPT with circular echo-planar trajectories^34^ are the most efficient; they work by interleaving spatial k-space readouts of echo-planar, spiral, circular encoding with the spectral acquisition to speed up the acquisition and cover more volume. However, these fast MRSI techniques usually have a limited spectral width and require more complex reconstruction procedures to correct strong off-resonance effects caused by echo-planar or spiral encoding. The PCSI method provides a different means of speeding up acquisition by using adaptive sampling in targeted ranges of the frequency domain. We compared these methods based on the parameters related to speed and spectral width, which are summarized in Table 1. When SNR is not considered in this comparison, PCSI is the most efficient of the five methods compared, based on the acquisition time per slice per average. Figure S6 shows that PCSI can generate well-defined spectra for a phantom or human, even with only one average. More averages can be used to improve the SNR when necessary.

All of the above methods can be further accelerated by using a parallel imaging or compressed sensing technique^35^,^36^,^37^,^38^. Acceleration has been demonstrated to achieve additional speed factors in phase and slice directions when parallel imaging techniques are used^12^,^16^,^17^,^18^,^39^. In the future, parallel imaging or compressed sensing techniques may be used with speed factors of 2 or more in conjunction with PCSI to acquire higher-resolution spectroscopic images when the SNR is sufficient.

The PCSI method has great flexibility for sampling the frequency domain. In this study, we demonstrated a non-uniform sampling scheme that covered only three metabolite peaks (NAA, Cr, and Cho), which decreased the total acquisition time by a factor of 2.5. If we cover only a single metabolite, the acquisition time can be further reduced by a factor of 3 to 5 to achieve the total acquisition in less than 1 min. PCSI may thus become suitable for studying the dynamic changes of certain metabolites.

### J-coupled metabolites

J-coupled metabolites (e.g., Glu and mI) have low signals in the conventional spectrum. With decreasing TE, the spectral signal increases considerably and makes the measurement of J-coupled metabolites feasible^40^,^41^,^42^. The PCSI method has a very short TE (1.2 ms). The PCSI signal is from a steady state mixed with shorter and longer TE signals. Even though the effective TE may be longer than the actual TE, the effective TE in PCSI may still be shorter than the minimum TE (30 ms) of PRESS SVS on the scanner. In Fig. 4, the mI peak in the PCSI spectrum is much higher than that in the SVS spectrum. Figure 4 clearly demonstrates that the PCSI method has some advantages with regard to higher signals in J-coupled metabolites.

Besides peak splitting of coupled metabolites, J coupling also causes phase evolutions that lead to peak and baseline distortion, thereby making measurements more difficult in a conventional time-resolved spectrum^43^,^44^. The PCSI method acquires data in the frequency domain. All metabolite peaks are acquired separately and should have consistent phases, because PCSI synchronizes the phase of each peak by changing RF cycled phase to match its frequency. The synchronized phases for all the frequencies in one spectrum may further increase the signal of coupled metabolites, which may make PCSI an ideal tool for measuring J-coupled metabolites. This will be investigated in future studies.

### Other advantages

PCSI offers other advantages over conventional MRSI. First, PCSI is less prone to problems such as unreliable spectra at the periphery of the brain due to OVS or a limited coverage of PRESS excitation. Second, by using an ultra-low flip angle, PCSI reduces the SAR in humans thousands of times compared to conventional spectroscopy sequences. PCSI also does not require spatial and water suppression, leading to an even greater reduction in the SAR^19^,^21^. Finally, the precise manual placement of OVS bands is often highly operator dependent and is poorly reproducible, even by the same operator, resulting in greater inter-subject variability^45^. With its intrinsic water and fat suppression, PCSI avoids the above issues, reduces subjective variability, and greatly simplifies the scanning procedures, thereby more closely resembling a routine clinical imaging procedure.

### Factors influencing PCSI

The T_1_/T_2_ ratio and flip angles are the main factors influencing PCSI. The relative heights of the peaks in Figs 3, 4 and 6 were somewhat different for PCSI and SVS, possibly reflecting the different mechanisms of data acquisition. The PCSI signal is from the bSSFP sequence, which is a steady state with mixed magnetizations across a broad range of TE values. The relative heights are determined mainly by factors such as T_1_/T_2_ and flip angles according to the signal equation [S8]. By comparison, the SVS signals are from a spin-echo sequence, and the relative heights are determined by T_2_ and TE.

The distribution of metabolite signals in Fig. 8 is not entirely consistent between chemical shift imaging (CSI) and PCSI, possibly as a result of T_1_/T_2_, flip angle, and B_0_ field inhomogeneity. In addition, there are still small differences between repeated measures in Figs 7 and S7. More robust results may be achieved by further optimizing the acquisition scheme with more dense sampling around the targeted peaks (e.g., with a step of 0.5° or less).

One definition of the SNR in spectroscopy is the peak height divided by the noise^40^. For the PCSI and CSI methods, the SNR was computed from a single voxel (PCSI voxel size: 6.25 × 6.25 × 15 mm; CSI voxel size: 10 × 10 × 15 mm) from a metabolic phantom by using the NAA peak height and the standard deviation of the noise computed on the left side of the Cho peak, because the PCSI method has a limited frequency range for measuring noise. The SNR values for the PCSI and CSI methods were 19.8 and 90.9, respectively. However, it may not be rational to compare SNRs directly in this case, because the left side of the Cho peak may not be an ideal location for measuring noise due to possible metabolite signal at short TE, and several factors influence the SNR, such as the voxel size, number of averages, acquisition matrix, sampling density, and TE value, which are not identical for the two methods.

The quality of shimming is a key factor in spectroscopic imaging. The typical FWHM ranges from 12 to 20 Hz for *in vivo* brain shimming^40^. In this study, PCSI used an auto-advanced shimming to achieve a very sharp water peak with a 6.5-Hz width on the phantom. However, PCSI had a broader peak with a 35.7-Hz width in one human volunteer, compared to the SVS peak width of 17.5 Hz in a much smaller ROI. Therefore, the quality of the PCSI spectrum may be improved by using advanced shimming techniques (e.g., higher-order shimming), which can approach a width of 15 Hz or less in humans^46^,^47^.

### Limitations and future applications

The PCSI method has some limitations. PCSI has a smaller frequency spectral window than does conventional MRSI and requires prior knowledge of metabolite location to speed up the acquisition using non-uniform sampling. Therefore, the PCSI method is suitable for studying metabolite signals in a known spectral range but may not be optimal for exploring new metabolites. Prior knowledge of the specific spectrum is, therefore, required in order to develop a specialized PCSI protocol for targeted metabolites. However, this may not be a problem in most clinical scenarios. In addition, because of the signal dependence on the flip angle, PCSI is sensitive to B_1_ inhomogeneity, which may be the main factor causing the variations in the parametric map shown in Fig. 8. Therefore, a body transmit coil should be used instead of a surface transmit coil to reduce the effects of B_1_ inhomogeneity. If necessary, B_1_ correction or parallel transmit can be used to further reduce these B_1_ inhomogeneity effects^48^.

The PCSI method has great potential for other applications. First, PCSI could be implemented in 3D imaging, which could have a higher SNR and efficiency because each 3D measurement requires only one steady state. Second, the PCSI can be used in multinuclear spectroscopic imaging, such as fluorine and sodium imaging. In addition, whereas time-resolved spectroscopy has a clear advantage in single-voxel spectroscopy, the PCSI method offers a greater advantage in 2D or 3D high-resolution spectroscopic imaging, in which the number of spectral points is much smaller than the number of conventional phase encoding steps. We have successfully acquired 32 × 32 and 64 × 64 images; a higher-resolution spectroscopic image with a 128 × 128 matrix may be obtainable in the future if high-order gradient shimming, superconductor coils, and parallel RF transmission techniques can be used with this PCSI technique.

## Methods

The theory and simulations are presented in the Supplementary Information.

### Protocol design

The protocol parameters must be precisely selected based on prior knowledge of the targeted metabolite signal. In contrast to conventional MRSI, PCSI does not require additional water- and fat-signal suppression. In PCSI, water and fat signals are intrinsically suppressed, because the very sharp response function suppresses signal that occurs far from the selected frequency, as shown in Fig. 1b. To suppress both water and fat, appropriate values of the protocol parameters, such as the TR and flip angle *α*, had to be selected to optimize the suppression by positioning the water and fat peaks as closely as possible, using the periodic property of the response function. To do this, the water and fat peaks had to be put into two consecutive frequency periods, as shown in Fig. S3a, so that the fat peak could wrap around to the water period as the red peak. The period length should be similar to the water-fat chemical shift for this configuration. The average chemical shift between body fat and water is approximately 3.35 ppm^49^, or approximately 413 Hz on a 3T scanner. Therefore, a TR of 2.4 ms was selected so that the period of the response function was 417 Hz, the closest value to the water-fat shift of 413 Hz. Period II was selected for the acquisition window because it included all the metabolite and water peaks. The fat peak was in period I and wrapped around to period II as an inverted red peak, as shown in Fig. S3a. In this case, both the water and fat peaks were located at the end of period II. The response function is different in each period, being positive for period II and negative for the two neighboring periods (I and III) in Fig. S3b. This is why the fat peak in period I was inverted and projected onto period II in Fig. S3a. To place the targeted metabolite at the center of the acquisition window, the system frequency was decreased by a certain amount (e.g., 200 Hz) to shift the water peak to one side of period II in Fig. S3a. For TR = 2.4 ms, T_1_ = 1300 ms, and T_2_ = 250 ms, the optimal *α* and the maximum amplitude are 0.24° and 0.22, respectively, according to equation [S11].

### Accelerated non-uniform frequency sweeping

When uniform sampling was used in the whole phase cycle (a frequency period) with a step of 1°, PCSI required approximately 11 min with an acquisition matrix 32 × 32 (360 measurements, TR = 2.4 ms, 23 averages). By using non-uniform sampling, we can sample densely in the ranges of target metabolites and sparsely in other ranges to reduce the acquisition time. In this study, the positions of three target metabolites, NAA, Cr, and Cho, in the spectrum were calculated in order to select the specific ranges for dense sampling. NAA is located near −115°, Cr is near −6°, and Cho is near 13°, with the water peak being shifted to 200 Hz on the 3T scanner. Therefore, the dense sampling windows were selected in the ranges (−142°, −100°) and (−27°, 40°) with a step of 1°, and the step in other ranges was selected as 10° (Fig. S4). The complete sample range of the cycled RF phase was chosen as −200° to 250°, which led to a total of 143 measurements and a total acquisition time of 4 min 28 s. The acquisition time was decreased by a factor of 2.5 by using this non-uniform sampling method when compared to a uniform sampling scheme with 360 measurements. With more advanced non-uniform sampling schemes, the acquisition time may be further reduced to less than 1 min in the future.

### Data acquisition

#### Phantom study

All studies were performed on a Siemens 3T Prisma MRI scanner (Siemens Medical Solutions, Erlangen, Germany). A phantom filled with a brain-mimicking solution containing the metabolites NAA, Cr, and Cho was used to investigate the feasibility of the PCSI method. The PCSI data acquisition used a modified bSSFP sequence based on a TrueFISP sequence, in which the cycled RF phase changed for each measurement. A 64-channel head coil was used in the image acquisition to ensure the quality of the data. In the experiments, a single axial slice was selected, and shimming with the advance mode was performed to achieve the best field homogeneity. After shimming, the system frequency was manually decreased by 200 Hz to shift the water peak toward the end of the frequency acquisition window. The protocol parameters were as follows: TR = 2.4 ms; TE = 1.2 ms; flip angle *α* = 0.3°; 40 dummy pulses for the preparation of the steady state; 32 × 32 acquisition matrix; in-plane resolution 6.25 × 6.25 mm; slice thickness 15 mm; bandwidth 1116 Hz; 23 averages; 143 measurements using a non-uniform sampling scheme of the cycled RF phase; and total time approximately 4 min 28 s. A flip angle of 0.3° was used because the actual flip angle may be smaller than the prescribed angle as a result of the electron screening effect.

In addition, a PCSI data set with uniform sampling (a step of 1°, 360 measurements) was acquired in 11 min 12 s. The short TE spectrum was acquired on a phantom by using the SVS protocol with a minimum TE of 30 ms. The PCSI signals of J-coupled metabolites were compared with those in the short-TE SVS spectrum. To test the limits of PCSI, a higher-resolution dataset was acquired with a 64 × 32 acquisition matrix, an interpolated in-plane resolution of 3.75 mm × 3.75 mm, and a slice thickness of 15 mm. The total acquisition time was 11 min 12 s. Half of the coil channels were turned off because of the limited memory of the computer used for processing the data. Finally, the above non-uniform protocol was performed three times on the phantom to test its repeatability.

#### Human studies

Field inhomogeneity is a more serious confounding variable in studies of the human brain than in those performed on a uniform phantom, making shimming a more critical issue. A single tilted axial slice was selected in the superior brains of two healthy volunteers and a patient with multifocal anaplastic astrocytoma of the brain to demonstrate the feasibility of PCSI. The protocol was the same as that used in the phantom study, except for the flip angle and the frequency shift. In the human studies, based on our experience, a flip angle of 0.5° was used to obtain a better signal. The frequency shift was reduced to 190 Hz because of the much broader water peak. Data were acquired from the first volunteer by using a 64-channel head coil (Fig. 6). Data from the second volunteer (Fig. 7) and the patient (Fig. 8) were acquired using a 20-channel head coil to demonstrate the feasibility and reproducibility.

For comparison, spectra from a single voxel were acquired in both phantom and human studies by using the manufacturer’s SVS sequence with PRESS preparation. The protocol TE was 135 ms, the TR was 2000 ms, and the voxel size was 20 × 20 × 20 mm. Water suppression was used to improve the SNR of the spectra. The total acquisition time was 4 min 24 s. The conventional CSI data were acquired from a patient with the following protocol parameters: TE/TR = 135/1700 ms; voxel size = 10 × 10 × 15 mm; 10 × 10 acquisition matrix; and total time of 8 min 7 s. These studies were approved by the institutional review board of St. Jude Children’s Research Hospital, and written informed consent was obtained from the volunteer, patient, parent, or guardian as appropriate. All the experiments were carried out in accordance with the approved guidelines.

#### Image reconstruction and post-processing

The measurement data in k-space were saved on the scanner and transferred to a Linux workstation. The image was first reconstructed by using a fast Fourier transform for each channel of the head coil. Phase correction based on the water peak was performed for each voxel to convert the real profile to a pure absorption shape, which ensured that the phases from different coil channels were consistent for each voxel. After phase correction, images from different channels can easily be combined by using a weighted summation based on their magnitude. Because of the field inhomogeneity, the position of the water peak varied for the different voxels in the image. The peaks were shifted in order to align them for subsequent processing. The baseline correction was performed by using polynomial fitting for the magnitude and phase of the profile without the metabolite signal. The profile then subtracted the fitted baseline to obtain the spectrum in units of Hertz. The spectrum was then converted to the final spectrum in parts per million (PPM). To quantify the spectrum, it was fitted using three Lorentzian functions. Normalization was performed by dividing the signal by a factor that was computed using an empirical form: the square root of the cube of the filtered sensitivity map. With the fitted spectrum, the different parameters (e.g., the amplitude and position) related to each peak could be extracted for further processing. After quantification for each voxel, the parametric maps were generated, registered, and overlaid on a high-resolution T_2_-weighted image. The signal-processing procedure is summarized and shown in Fig. S5; in the future, it may allow real-time metabolic maps to be generated directly on the MRI scanner without the need for manual intervention by a technician.

## Additional Information

**How to cite this article**: Guo, J. *et al*. Fast frequency-sweep spectroscopic imaging with an ultra-low flip angle. *Sci. Rep.* **6**, 30066; doi: 10.1038/srep30066 (2016).

## Supplementary Material

Supplementary Information

## Figures and Tables

**Table 1:** Comparison of several fast MR spectroscopic imaging methods.

Methods	Matrix	Spectral BW (Hz)	Total time (min:s)	Slice#	Time per slice (min:s)	Average#	Time per slice per average (s)
CSI*	10 × 10	1200	8:07	1	8:07	3	162
EPSI^14^	32 × 32	488	1	1	1	1	60
PEPSI^12^	32 × 16	390	2:08	1	1	4	32
SI-CONCEPT^34^	24 × 24	1250	5:54	1	5:54	16	22
Spiral-SI^13^	18 × 18	400	21	10	2:06	6	21
PCSI*	32 × 32	417	4:28	1	4:28	23	12

^*^Represents the data from this study. BW: bandwidth.
