# Supplementary material for: Fast frequency-sweep spectroscopic imaging with an ultra-low flip angle
Source: Sci Rep. 2016 Jul 21;6:30066. doi: 10.1038/srep30066 (PMC4954958; doi:10.1038/srep30066)
Supplement: Supplementary Information [file srep30066-s1.pdf]

Fast frequency–sweep spectroscopic imaging with an ultra-low flip angle

Junyu Guo, Ph.D.[1], Zoltan Patay, Ph.D, MD[1], & Wilburn E. Reddick, Ph.D[1]

[1]Department of Diagnostic Imaging, St. Jude Children's Research Hospital, Memphis, Tennessee 38105

Supplementary Information (SI)

SI Theory

Steady-State Signal Equation

A steady-state signal equation was derived for the bSSFP sequence with a cycled RF phase φ, which was based on the scheme diagrammed in Fig. S1a. The matrix representation theory was used to derive the steady-state magnetization[1,2]. As nuclear magnetization precession is clockwise, rotational matrices with an angle α were defined as follows:

$$R_x^-(\alpha) = \begin{bmatrix} 1 & 0 & 0 \\ 0 & \cos\alpha & \sin\alpha \\ 0 & -\sin\alpha & \cos\alpha \end{bmatrix},$$

$$R_y^-(\alpha) = \begin{bmatrix} \cos\alpha & 0 & -\sin\alpha \\ 0 & 1 & 0 \\ \sin\alpha & 0 & \cos\alpha \end{bmatrix}, \qquad [S1]$$

$$R_z^-(\alpha) = \begin{bmatrix} \cos\alpha & \sin\alpha & 0 \\ -\sin\alpha & \cos\alpha & 0 \\ 0 & 0 & 1 \end{bmatrix}$$

For a component with off-resonance frequency of $\Delta f = 2\pi\Delta\omega$, the precession angle at time of echo (TE) is $\theta = 2\pi \cdot \Delta f \cdot TE$, as shown in Fig. S1b. The magnetization at the $(n+1)^{th}$ echo, $M_{n+1}^{\Delta\omega}$, is calculated from M_+, the magnetization just after the $(n+1)^{th}$ RF pulse in Fig. S1a:

$$M_{n+1}^{\Delta\omega} = E_A R_Z^-(\theta) M_+ + E_B \qquad [S2]$$

$$E_A = \begin{bmatrix} e_2 & 0 & 0 \\ 0 & e_2 & 0 \\ 0 & 0 & e_1 \end{bmatrix}, E_B = (1 - e_1) M_0 = \begin{bmatrix} 0 \\ 0 \\ 1 - e_1 \end{bmatrix}, e_1 = e^{-\frac{TE}{T_1}}, \; e_2 = e^{-\frac{TE}{T_2}}, M_0 = \begin{bmatrix} 0 \\ 0 \\ 1 \end{bmatrix}, \qquad [S3]$$

where T_1 is the spin-lattice relaxation time and T_2 is the spin-spin relaxation time. The matrices E_A and E_B represent the relaxation process. The matrix $R_z^-(\theta)$ represents a precession process of an off-resonance component with $\Delta\omega$ during TE.

By using equation [S2], the magnetization is evolved from $M_n^{\Delta\omega}$to $M_{n+1}^{\Delta\omega}$ as follows:

$$\mathrm{M}_{-} = \mathrm{E_A R_Z^-(\theta) M_n^{\Delta\omega}} + \mathrm{E_B} \ ,$$

$$\mathrm{M}_{+} = \mathrm{R_x^-(\alpha_{n+1}) M_-} \ , \qquad \text{[S4]}$$

$$\mathrm{M_{n+1}^{\Delta\omega}} = \mathrm{E_A R_Z^-(\theta) M_+ + E_B}.$$

To simplify the form in equation [S4], the rotation axis of the $(n+1)^{th}$ RF pulse is selected as the X axis, and the second equation in equation [S4] then becomes simpler without the term of the cycled RF phase φ. The cycled RF phase is implicitly included in M_n, which is included in the steady-state equation below.

When reaching the steady state, the relationship of $M_n^{\Delta\omega}$to $M_{n+1}^{\Delta\omega}$ should be as follows:

$$\mathrm{M_{n+1}^{\Delta\omega}} = \mathrm{R_Z^-(\varphi) M_n^{\Delta\omega}}, \qquad \text{[S5]}$$

where φ is the change in the RF phase from the nth RF to the $(n+1)^{th}$ RF pulse. φ is a constant for each measurement (or each image).

By solving equations [S4] and [S5], the steady-state magnetization is derived as

$$\mathrm{M_n^{\Delta\omega}} = \frac{\mathrm{E_A R_Z^-(\theta) R_x^-(\alpha_{n+1}) E_B + E_B}}{\mathrm{R_Z^-(\varphi) - E_A R_Z^-(\theta) R_x^-(\alpha_{n+1}) E_A R_Z^-(\theta)}}. \qquad \text{[S6]}$$

The complex form of the transverse magnetization in equation [S6] becomes

$$\mathrm{M_{xy}^{\Delta\omega}} = \frac{\mathrm{e_2(1-e_1^2)\left(e_2^2 e^{-i\theta} - e^{-i(\varphi-\theta)}\right)\sin\alpha}}{\mathrm{e_2^2\cos(\varphi-2\theta)(1+\cos\alpha)(1-e_1^2) + (e_1^2 - e_2^4)\cos\alpha + e_1^2 e_2^4 - 1}}, \qquad \text{[S7]}$$

and the magnitude of the transverse magnetization becomes

$$\left|M_{xy}^{\Delta\omega}\right| = \frac{e_2(1-e_1^2)\sin\alpha\sqrt{e_2^4 - 2e_2^2\cos(\varphi-2\theta)+1}}{1-e_2^2\cos(\varphi-2\theta)(1+\cos\alpha)(1-e_1^2)-(e_1^2-e_2^4)\cos\alpha - e_1^2e_2^4}. \qquad \text{[S8]}$$

Equation [S8] was verified by comparing the results obtained in the following two special cases with the previously reported results.

For $\varphi, \theta = 0$, equation [S8] becomes

$$\left|M_{xy}^{0}\right| = \frac{e_2(1-e_1^2)\sin\alpha}{1-(e_1^2+e_2^2)\cos\alpha + e_1^2e_2^2}, \qquad \text{[S9]}$$

and for $\varphi = \pi, \theta = 0$, which is used in most SSFP sequences, equation [S8] becomes

$$\left|M_{xy}^{0}\right| = \frac{e_2(1-e_1^2)\sin\alpha}{1-(e_1^2-e_2^2)\cos\alpha - e_1^2e_2^2}. \qquad \text{[S10]}$$

Equations [S9] and [S10] are consistent with equations [14.23] and [14.24] described by Bernstein et al.[3].

For spectroscopic imaging, the signal-to-noise ratio (SNR) is an important factor. To achieve the maximum signal, an optimal RF flip angle α was calculated for the maximum magnetization $\left|M_{xy}^{0}\right|$ for $\varphi, \theta = 0$ by using equation [S9].

$$\alpha_{max} = \arccos\left(\frac{e_1^2+e_2^2}{1+e_1^2e_2^2}\right)$$

$$M_{xy_{max}} = \frac{e_2(1-e_1^2)}{\sqrt{1-e_1^4-e_2^4+e_1^4e_2^4}} \qquad \text{[S11]}$$

This optimal α is the same for different φ values, because the magnetization profiles are shifted only as φ changes in Fig. S2c.

Simulation

Simulations were performed to demonstrate the feasibility of this PCSI method. In equation [S8], with $\varphi = 0$, the transverse magnetization is a periodic function of the precession angle θ, which corresponds to an off-resonance frequency. The period of this function is $2\theta_T = 2\pi$, which corresponds to an off-resonance frequency range of $\Delta f_T = 1/TR$. The optimal flip angle depends on the TR, T_1, and T_2 values of the metabolite and can be calculated using equation [S11]. This optimal flip angle may ensure the best SNR for the spectrum. The optimal flip angle was computed to be 0.24° for TR = 2.4 ms, $\mathrm{T_1} = 1300\ \mathrm{ms}, \mathrm{T_2} = 250\ \mathrm{ms}$, which were selected on the basis of the reported values of three targeted metabolites on a 3T scanner[4,5].

The magnitude profiles of the transverse magnetization were computed for different flip angles (0.24°, 1°, 10°, 30°) using the same values of TR, T_1, and T_2 as above. Magnitude profiles for different flip angles, computed using equation [S8], are shown in Fig. 1a. The conventional bSSFP sequence uses a large flip angle (e.g., 30°) to generate a magnetization profile with a plateau spanning a wide range of frequencies and a crevice in a small range of frequencies, as shown in Fig. 1a. The bSSFP sequence excites magnetization in the plateau range of frequencies to obtain MR images, but band artifacts may occur because of the small range of frequencies in the crevice [6]. As the flip angle decreases to less than 1°, the crevice becomes a sharp peak and the plateau becomes a wide U-shaped valley (Fig. 1a). The profiles computed from

equation [S7] are shown in Fig. 1b, in which the real part is a pure absorption component and the imaginary part is a dispersion component. The sharp, narrow peak indicates that magnetization resonates in only a very small range of frequencies (Fig. 1b). The peak width (the full width at half maximum [FWHM]) of the real part of the magnetization profile is very small, within the range of 1.9 to 5.5 Hz, with a time of repetition (TR) of 2.4 ms and flip angles of 0.24° to 1.0°.

In equation [S8], the magnetization is a periodic function of $\varphi - 2\theta$, which confers a relationship between the cycled RF phase φ and the precession angle θ as follows:

$$\varphi = 2\theta = 2\pi \Delta f TR$$
$$\Delta f = \frac{\varphi}{2\pi TR} \qquad [S12]$$

Equation [S12] shows the conversion between the cycled RF phase and the off-resonance frequency. This relationship is the foundation of this PCSI technique, which demonstrates how to choose the sweeping frequency by using the cycled RF phase φ. For each image acquisition, the real and phase profiles of magnetization were shifted by changing the cycled RF phase φ, as shown in Figs. S2a and S2b. However, the real profile is not a pure absorption component, as the cycled RF phase is shifted away from zero. Besides shifting in the frequency direction, there is an additional overall phase shift of $\varphi/2$ added to the phase profiles. To convert the real profiles to pure absorption functions requires the phase profile to shift only along the frequency direction with no additional overall phase shift. After correcting the overall phase shifting, the consistent real and phase profiles, shown in Figs. S2c and S2d, can serve as good response functions for spectroscopic imaging.

SUPPLEMENTAL REFERENCES

1. Woessner, D. E. Effects of diffusion in nuclear magnetic resonance spin-echo experiments. *J. Chem. Phys.* **34**, 2057−2061, doi:10.1063/1.1731821 (1961).
2. Jaynes, E. T. Matrix treatment of nuclear induction. *Physical Review* **98**, 1099−1105 (1955).
3. Bernstein, M. A., King, K. F. & Zhou, X. J. *Handbook of MRI Pulse Sequences*, 593 (Elsevier, 2004).
4. Mlynarik, V., Gruber, S. & Moser, E. Proton T-1 and T-2 relaxation times of human brain metabolites at 3 Tesla. *NMR Biomed.* **14**, 325−331, doi:10.1002/Nbm.713 (2001).
5. Haacke, E. M., Brown, R. W., Thompson, M. R. & Venkatesan, R. *Magnetic Resonance Imaging: Physical Principles and Sequence Design*, 135 (Wiley-Liss, 1999).
6. Hargreaves, B. A., Vasanawala, S. S., Pauly, J. M. & Nishimura, D. G. Characterization and reduction of the transient response in steady-state MR imaging. *Magn. Reson. Med.* **46**, 149−158, doi:10.1002/Mrm.1170 (2001).

SUPPLEMENTAL FIGURES

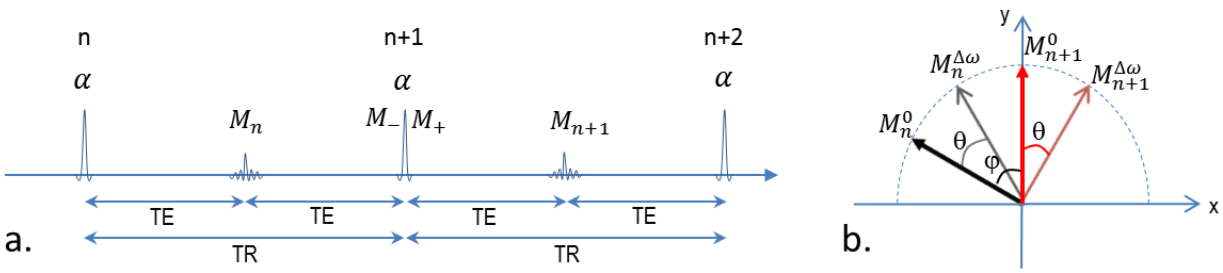

Figure S1. Diagrams of scheme and precession using balanced steady-state precession (bSSFP). (a) The scheme diagram includes two TR periods and three RF pulses with flip angle α and time of echo (TE). (b) Precession diagram from M_n to M_{n+1} for isochromat with off-resonance frequency of 0 and $\Delta\omega$.

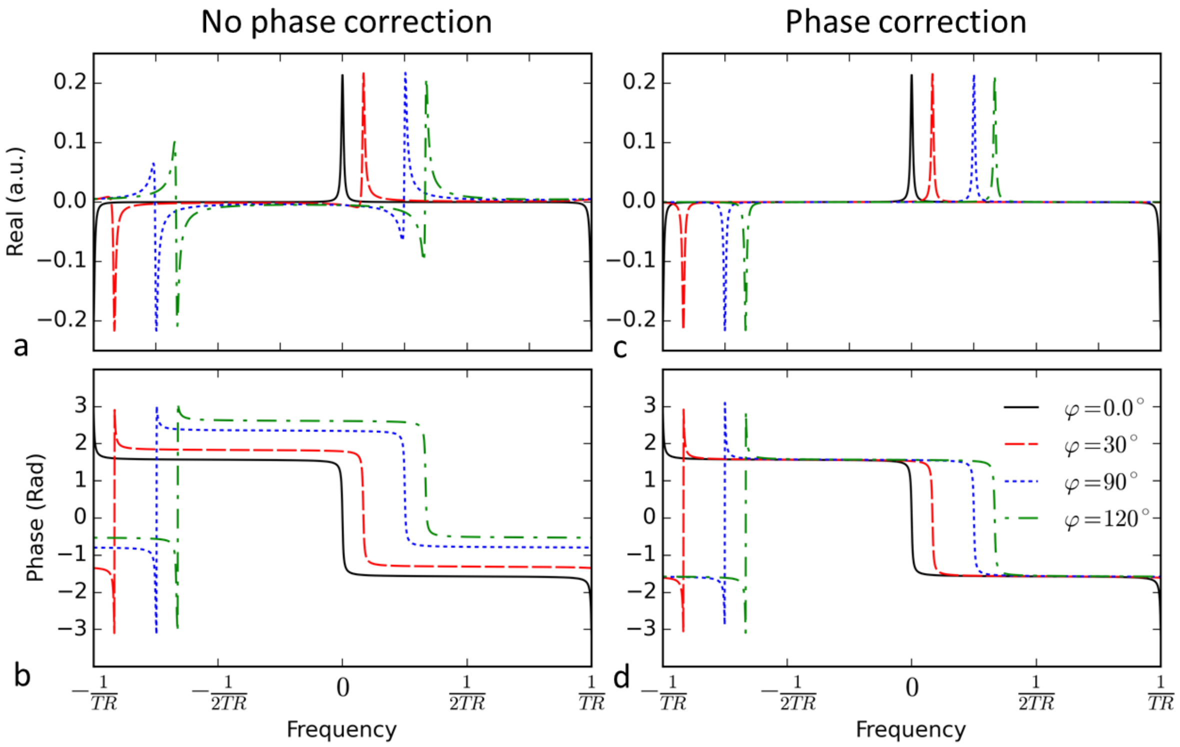

Figure S2. Real components and phase shifting of magnetization as the cycled RF phase (φ) increases, computed using equation [S7]. (a and b) Magnetization profiles without overall magnetization phase correction for the cycled RF phase. (c and d) Magnetization profiles with overall phase correction to ensure the phase shifts only along the frequency direction.

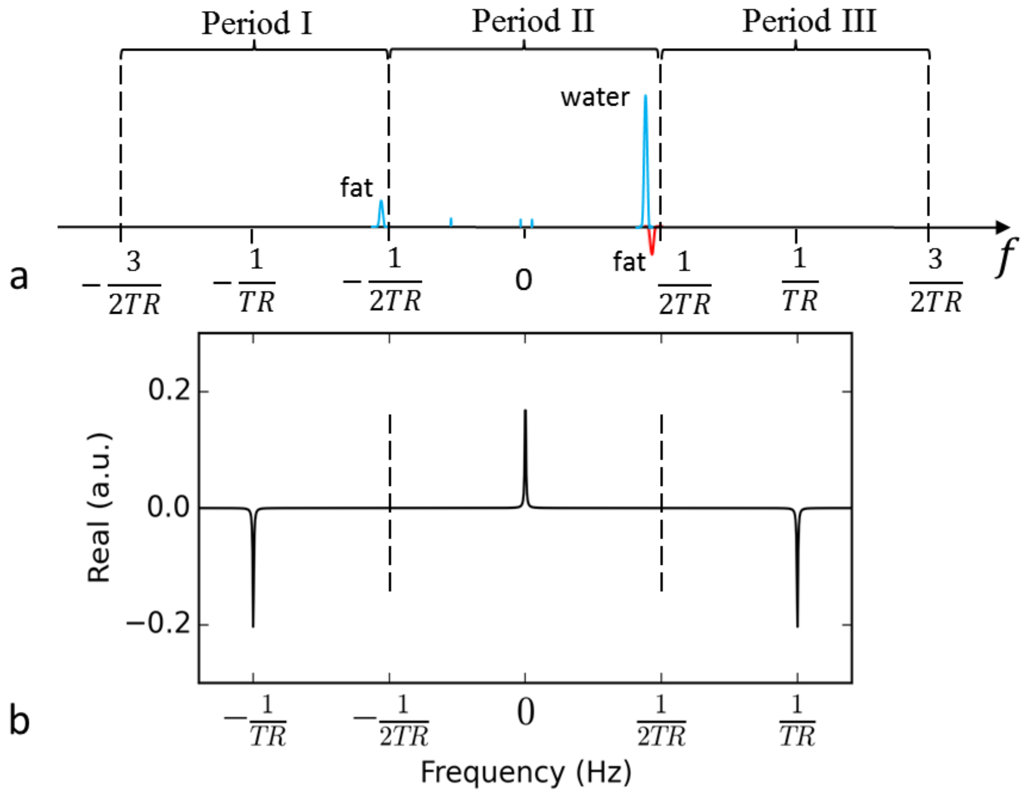

Figure S3. (a) Diagram of acquisition window selection. Period II was selected for the acquisition window so that the water peak would be located at the left end and the three small metabolites (NAA, Cr, and Cho) would be located near the center of the acquisition window period. (b) The response function computed using equation [S7] has a different direction for each period. The fat peak in period I is inverted and wrapped around to period II as the inverted red peak.

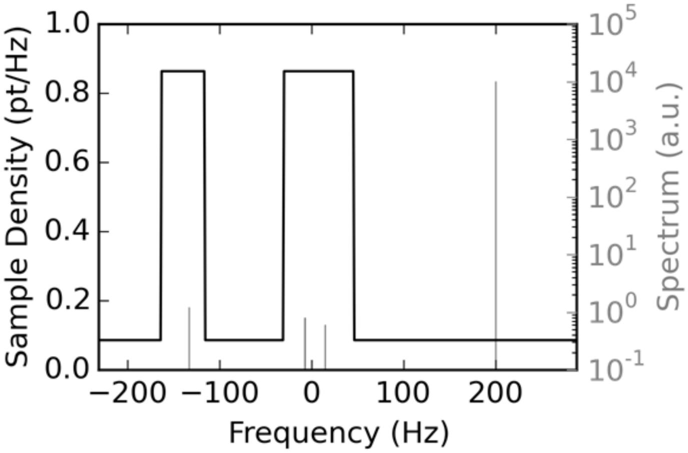

Figure S4. Sample density of non-uniform PCSI. A higher sample density was chosen for the range with the targeted metabolites (NAA, Cr, Cho), shown as gray vertical lines. From left to right, the gray lines represent NAA, Cr, Cho, and water. The water peak was assumed to shift to 200 Hz.

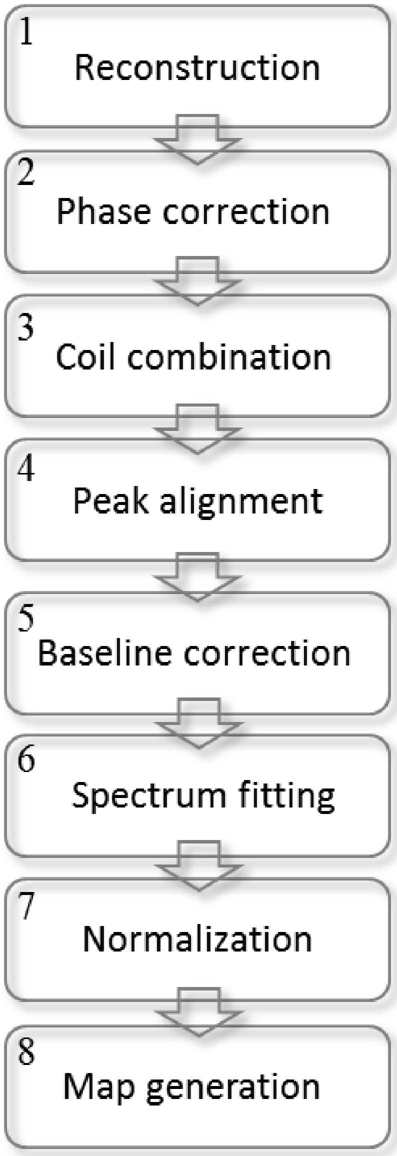

Figure S5. Diagram of the signal processing pipeline.

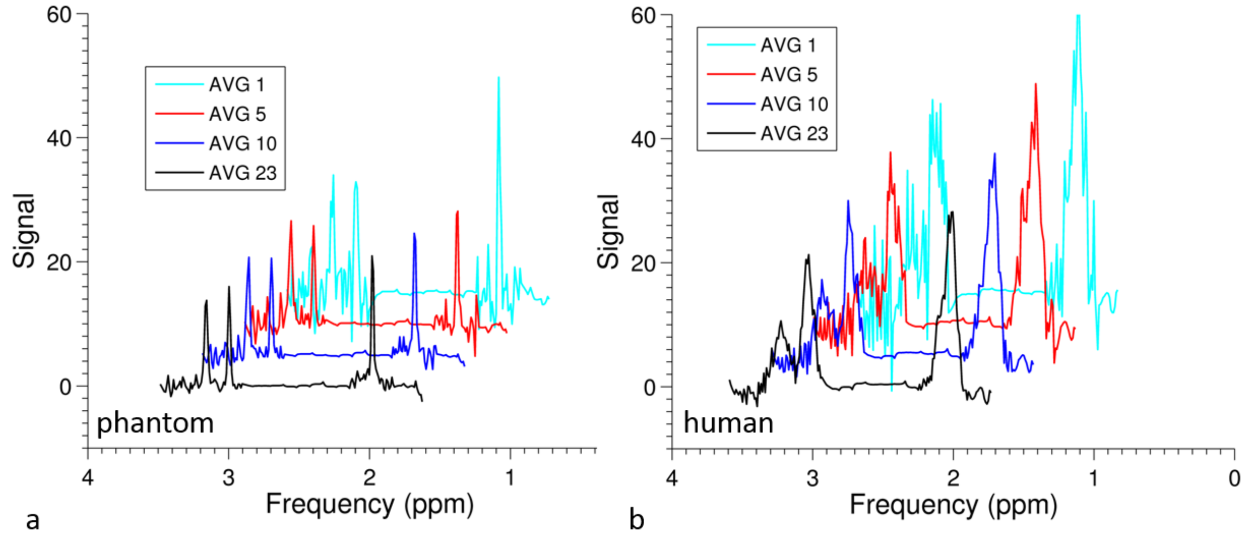

Figure S6. Spectra of a phantom and a human obtained using different numbers of averages. (a) Phantom spectra for 1, 5, 10, and 23 averages. (b) In vivo spectra for the same number of averages. Both spectra were from an ROI with 3 × 3 voxels (18.75 × 18.75 × 15 mm). Compared with the spectra obtained with AVG 23, the other spectra were sequentially shifted to the right by 0.3 ppm and were shifted up by 5 for display purposes. AVG = average.

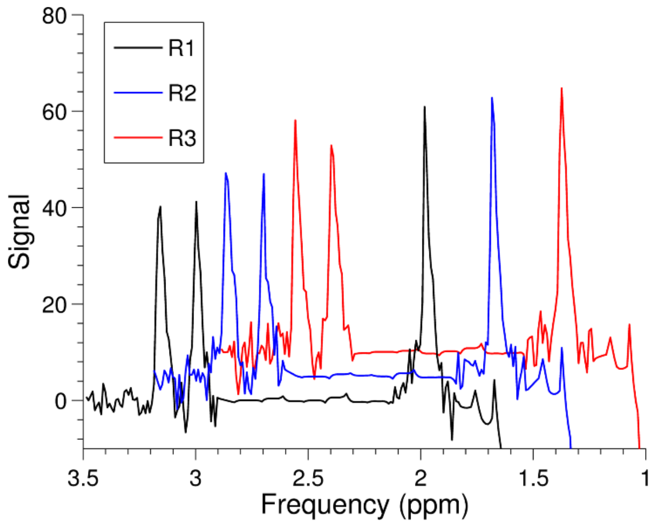

Figure S7. Three spectra obtained from a phantom to demonstrate the repeatability. All the spectra are from the same voxel (6.25 × 6.25 × 15 mm). R1, R2, and R3 represent three repeated measurements. The spectra were sequentially shifted to the right by 0.3 ppm and were shifted up by 5 for display purposes.
